# A framework for medical physics compensation in an academic department

**DOI:** 10.1002/acm2.14505

**Published:** 2024-09-09

**Authors:** David P. Gierga, Tiffany Zewe, Su Yoon, Thomas R. Bortfeld

**Affiliations:** ^1^ Department of Radiation Oncology Massachusetts General Hospital Boston Massachusetts USA; ^2^ Harvard Medical School Boston Massachusetts USA

**Keywords:** compensation, professional, salary

## Abstract

Compensation is a key component of career satisfaction and professional growth. A new compensation model was developed to provide a framework for career growth and a compensation ladder for medical physicists with clinical responsibilities in an academic radiation oncology department. The goals for the new model were: (1) create a market competitive plan to support recruitment and retention of top physics talent, (2) incentivize clinical effort, innovation, citizenship/professional service, and academic achievement, (3) provide compensation growth opportunities separate from medical school promotions, and (4) create consistent, transparent, and fair metrics applicable to all clinical physicists in the department. The model includes a base salary, and credits for board certification, clinical tier, leadership, and academic level. Further, metrics were developed to inform the clinical tier. Years of experience is not explicitly included in the model. The model was successfully implemented for clinical physicists in a relatively large academic radiation oncology department.

## 1 INTRODUCTION

Compensation is an important aspect of career planning and professional growth for medical physicists. A robust compensation plan will attract and retain talented people and incentivize activities consistent with the goals of the institution. Within radiation oncology, physician compensation is often tied to relative value units (RVUs)^1^ but current billing models do not adequately capture clinical physicists’ effort.^2^ The annual AAPM Professional Survey provides useful aggregate salary data^3^ and salary data are available for select public institutions, but to our knowledge, there is no existing literature describing the structure of a medical physics compensation model. We therefore present a model developed for clinical medical physicists in an academic radiation oncology department which we believe will be useful for medical physicists and radiation oncology leaders.

Prior to implementation of this new model in our department, physicist compensation roughly followed the AAPM salary scale based on years of experience, with additional stipends for American Board of Radiology (ABR) certification, departmental leadership responsibilities, and medical school academic promotions (Assistant Professor, Associate Professor, and Professor, for PhD‐level physicists only). There typically exists a range of interest and opportunity for clinical medical physicists to pursue academic promotion,^4^ so while there was a mechanism to reward more academically productive physicists, no specific pathway existed to reward those who were more clinically focused. The model described in this paper is intended to provide a framework for career growth and a compensation ladder for medical physicists with clinical responsibilities in an academic department.

## 2 METHODS

The goals for the new model were:

1. create a market competitive plan to support recruitment and retention of top physics talent,
2. incentivize clinical effort, innovation, citizenship and professional service (e.g., internal and external committee service, teaching, and mentorship) and academic achievement,
3. provide compensation growth opportunities separate from medical school promotions, and
4. create consistent, transparent, and fair metrics applicable to all clinical physicists (either MS or PhD) at both main campus and network locations.

In constructing the model, we consulted with medical physics groups from peer institutions as well as publicly available salary data.^5^, ^6^ Taken together, these goals were meant to create a platform for salary growth for both clinically focused and academically active physicists, and reward productive performance above and beyond basic clinical service based on more than simply years of experience.

The model was created for a group of medical physicists at a large academic hospital that values clinical care, research, and teaching. The model parameters and structure were developed jointly by physics and departmental administrative leadership, with input and support from physician and institutional leadership, and Human Resources. Model values were iterated to achieve a consensus agreement between improved compensation and feasible budget values.

There were 34 physicists included in the model, including three network locations, with years of experience ranging from 1 to 36 years. Physics faculty with research responsibilities but no clinical effort was included in the model.

## 3 RESULTS

The compensation plan is based on a salary increment unit Δ$. The components are:

1) Base salary: 10Δ$

The base salary is independent of the number of years of experience, plus credits for

2) ABR certification: 0 or 2Δ$
3) Clinical tier: 0, 1, 2 or 3Δ$

The clinical tier credit is based on the components shown in Table 1, with salary increases associated with Tiers II–IV. Consideration for movement to the higher tier is dependent on sustained, proven performance at the higher tier's expectations, but not every item needs to be achieved to advance to the next level. Tiers are assigned by the Physics Division Chief and Director of Clinical Physics with the expectation that progress through the tiers would be discussed with each physicist at least annually.

4) Leadership: 0, 1, 2, or 3Δ$

**TABLE 1:** Tier metrics.

a) Tier I	
Area	Examples of metrics
Clinical productivity	Non‐ABR or ABR certified Does clinical tasks with guidance
Project productivity	Participation in project(s) with guidance and close supervision
Citizenship and professional service	Participation in departmental committee(s) Participation in DEI activities Involvement in administration and institutional service ○ Policies ○ Guidelines ○ Protocol management Involvement in committees of professional societies (e.g., AAPM), primarily as member

b) Tier II	
Area	Examples of metrics
Clinical productivity	Independently manages clinical tasks
Project productivity	Active participation in project(s) Independent execution of project tasks Reach project milestones Timely completion by given deadlines
Citizenship and professional service	Participation in departmental committee(s) Mentorship Active participation in DEI activities Teaching: for example, Suffolk, Harvard learners (teaching of Harvard learners is expected as part of the academic mission) Administration and institutional service ○ Policies ○ Guidelines ○ Protocol management Involvement in committees of professional societies (e.g., AAPM)

c) Tier III	
Area	Examples of metrics
Clinical productivity	Supervises clinical tasks Takes on new clinical roles/expanded skill set (above and beyond basic clinical service) Has responsibility for clinical services (not including formal leadership role), software/hardware, and/or disease site Consistent track record of leading practice‐changing implementation of new technology or clinical procedures Clinical resource for other role groups
Project productivity	Active participation in projects Serve as project leader Successful execution of entire projects Proactive identification of areas for improvement Timely completion by given deadlines
Citizenship and professional service	Leadership of departmental committee(s), including DEI Mentor several peers Leadership role in teaching, for example, Suffolk, Harvard learners (teaching of Harvard learners is expected as part of the academic mission) Leading role in administration and institutional service ○ Policies ○ Guidelines ○ Protocol management Chair/co‐chair in committees of professional societies (e.g., AAPM)

d) Tier IV	
Area	Examples of metrics
Clinical productivity	Established local (or national) expert in one or more clinical areas Recognized (senior) clinical leader (separate from formal title) Lead clinical policy changes
Project productivity	Active participation in multiple projects Serve as project leader of major department‐wide projects Serve as project sponsor Successful execution of major projects (examples:) Identification of new projects/opportunities Timely completion by given deadlines
Citizenship and professional service	Leadership of major departmental committee(s) and creation of new committees, including DEI Mentor group of peers Leadership role in large teaching programs (teaching of Harvard learners is expected as part of the academic mission) Senior leadership role in administration and institutional service ○ Policies ○ Guidelines ○ Protocol management Chair/co‐chair in higher‐level committees (major workgroups, councils) of professional societies (e.g., AAPM)

Leadership credits are awarded for each of three levels: (1) service lead (e.g., brachytherapy or treatment planning lead), basic staff supervision (e.g., medical physics assistants), or educational leadership (e.g., residency program director), (2) assistant director or director, or 3) managing director.

5) Academic level: 0, 1, 2 or 3Δ$

Academic credits are based on promotion to Assistant, Associate, or Full Professor, with the promotion process governed by the guidelines of the Medical School.

**FIGURE 1:**
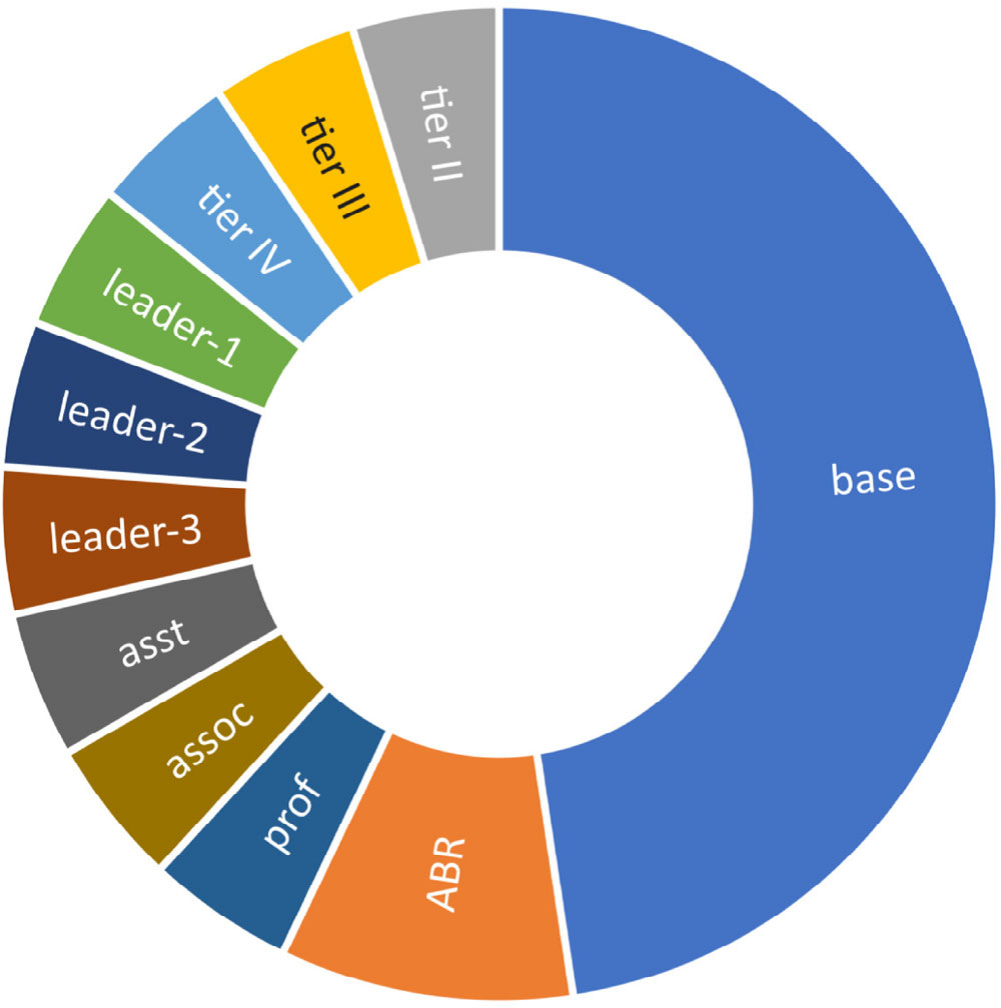
Relative contribution of compensation plan components. Base = base salary, 10Δ; ABR = credit for board certification, 2Δ; Asst, Assoc, and Prof = academic levels, Δ, Leader‐1, −2, −3: leadership credits, Δ; Tier II‐IV, clinical tier credits, Δ.

The various components of the compensation model are show in Figure 1. The salary increment unit **Δ**$ increases annually by the cost‐of‐living increase (y%) set by the hospital for all employees. Absent any other changes due to tier, leadership, or academic rank, an employee's salary would increase annually by y%.

The compensation model was implemented at the beginning of a fiscal year. Each physicist was assigned a tier rating and credits for the various model components. The modeled salary was compared to the existing salary (plus the annual cost of living increase) and higher of the two was selected. No physicist salaries were lowered as a result of the new compensation model.

## 4 DISCUSSION

A robust compensation plan has been designed those rewards and incentivizes the diversity of effort in an academic medical physics group and aligns with the clinical, research, and teaching goals of the department. To our knowledge, this is the first publication of a medical physics compensation plan in the literature. The model provides discrete objectives for both clinical and research‐based advancement and rewards citizenship/professional service activities both within the department and professional societies. Aside from academic promotion, the compensation structure for PhD‐ and MS‐level physicists is the same, which does result in a lower maximum compensation for MS‐level physicists. Note that physicists holding a Doctor in Medical Physics (DMP) degree can be employed by the hospital but would not be eligible for academic appointment per medical school guidelines. Compensation growth (through credits) is available for both clinical and academic achievements, which is notable as integrating a high clinical load with academic activities (e.g., publishing) can be challenging, just as clinical contributions may be less for the more research‐focused physicist.

Designing and implementing a new compensation plan can be challenging and the model described here includes several limitations. First, it should be recognized that although salary is certainly a major component, it is only one part of overall compensation. Institutional policies, including benefits and retirement plan options were not addressed in our model and are not within the department's control. Another limitation is that although metrics for advancing through the tiers are provided, a quantitative scoring system is not used, and the overall tier assessment is still somewhat qualitative. We do recommend that departments consider as many objective measures as possible when implementing a tier system. The tiers, furthermore, are broad, so progressing through the tiers is expected to take several years.

Two fictional examples are provided to better illustrate how the compensation model would be applied. First, consider Physicist‐1, who is an ABR‐certified PhD‐level physicist with academic rank of Assistant Professor based primarily on a publishing record consisting of mostly first‐author scholarship and a strong local reputation as a clinical physics expert. This physicist independently handles clinical tasks with little guidance from other physicists, has a sustained record of independent and productive contributions to recent clinical projects, actively mentors' physics and dosimetry students and peer physicists, and participates in several departmental and AAPM committees. Physcist‐1 is assigned Tier II, with a salary of 14Δ (10Δ base, 2Δ for ABR, 1Δ for Tier II, plus 1Δ for academic rank). Second, consider Physicist‐2, who is an ABR‐certified MS‐level physicist who is a “go‐to” person for peer physicists, physicians, dosimetrists, and therapists, provides supervisory guidance for clinical procedures, is the point person for various software tools, proactively and constructively identifies clinical gaps and has led successful high‐impact clinical projects, serves as a committee leader within the department and within AAPM, is a sought‐after mentor and teaches students/residents, and has formal leadership of a clinical physics service. Physicist‐2 is assigned Tier IV, with a salary of 16Δ (10Δ base, 2Δ for ABR, 3Δ for Tier IV, plus 1Δ for leadership).

The tier system provides opportunities and challenges. A major challenge is that physicist and leadership expectations of clinical tier level may not match, and the introduction of a “rating” system may require a culture adjustment within the group. First, physicists may receive a tier rating that indicates that their modeled salary may be lower than their actual salary. While their salary would not decrease, this feedback may be challenging. Second, a physicist's modeled salary may be above their current salary, but the physicist may expect a higher tier. The tier system, therefore, creates an opportunity for discussion between each physicist and leadership, including a review of specific milestones that could lead progressing to the next tier and therefore a salary increase. These discussions will be incorporated into the standard “Annual Career Conferences” required for all hospital professional staff. It is critical that the tier metrics be as clear as possible to ensure fairness and that all understand what is expected to reach the next level. Although years of experience certainly informs each physicist's knowledge and clinical skill, the compensation plan does not explicitly recognize year of experience, but rather specific and sustained contributions to the department's goals. It is not expected that physicists would regress to a lower tier, but it is possible. Clinical tiers, however, are evaluated over several years and short‐term fluctuations in effort or accomplishments should not affect the overall tier assignment.

The model is based on a discrete salary unit **Δ**$. Salaries are therefore within discrete bins, and it is expected that changes to the next bin will occur every few years due to tier changes, more (or less) leadership responsibilities, or academic promotions. This choice was made because salary changes (other than cost of living increases) must be budgeted, and it would be a challenge to re‐budget (and re‐justify) the salaries of all employees every year. The downside is that employees on either side of a bin may be slightly over‐ or underpaid. The value of **Δ**$ had to reflect this trade‐off between well‐defined and manageable salary metrics on the one hand, and a fine‐grained individual salary structure on the other.

The compensation model was set such that the department's salary scale compared favorably with the AAPM salary survey, with a trade‐off between increased compensation and internal budget constraints. The AAPM should be commended for publishing these helpful data, however, they are self‐reported and therefore, in our experience, weighted less by hospital administrators. Further, the data are retrospective, so simply prospectively matching the salary survey results in lagging behind the market. Publicly available salary data are also helpful^5^, ^6^ in benchmarking salaries. It should be noted that the newly released individualized AAPM salary calculator^7^ was not available when the model was developed but should be a useful tool in the future.

As noted previously, the total salary will increase annually by the published hospital annual cost of living increase. However, this may not be sufficient to keep pace with changes in the marketplace. We, therefore, plan to review and adjust the base and credit values after several years in collaboration with administration and budget permitting.

We further recognize that the model is most relevant to an academic setting, where physicists have varying clinical responsibilities, are encouraged to be active academically, and a promotion path is available through an affiliated medical school. The model was also developed in a therapy medical physics environment but could also apply to diagnostic or nuclear medicine groups.

One additional challenge could arise when onboarding new physicists to the group. When converting the group to a new compensation plan, leadership should be sufficiently informed to assign tier ratings appropriately, based on past performance, but this is not possible for experienced physicists joining the group. Since the compensation model has no explicit component for years of experience, the initial tier rating would be an estimate, based on an assessment of the incoming candidate's track record, with specific feedback provided on expectations and pathways for achieving tier progression. Academic level, where applicable, would need to be separately considered by the medical school's promotion committee. We believe, however, that the right approach is to hire for a specific role and set the clinical tier based on, as best as can be determined, the career achievements of the physicist, and not to have the existing budget influence the initial tier level. Lastly, although anecdotal feedback to the compensation changes was positive, physicist satisfaction was not explicitly measured.

## 5 CONCLUSION

A compensation model has been designed and implemented for a large academic medical physics group, providing a framework for salary growth associated with clinical and academic achievements, leadership, and citizenship.

## AUTHOR CONTRIBUTIONS

All authors contributed equally to this article.

## CONFLICT OF INTEREST STATEMENT

The authors declare no conflicts of interest.

## References

[1] Hogan J , Roy A , Karraker P , et al. Decreases in radiation oncology medicare reimbursement over time: analysis by billing code. Int J Radiat Oncol Biol Phys. 2022;114(1):47‐56. doi:10.1016/j.ijrobp.2022.05.018 35613687 PMC10077845

[2] Herman MG , Mills MD , Gillin MT . Reimbursement versus effort in medical physics practice in radiation oncology. J Appl Clin Med Phys. 2003;4(2):179‐187. doi:10.1120/jacmp.v4i2.2533 12777154 PMC5724472

[3] AAPM Professional Survey Report, Calendar Year; 2022.

[4] Harvard University Faculty of Medicine Handbook. https://facultyhandbook.hms.harvard.edu/accessed 1/31/24

[5] Accessed 7/7/2023. https://openpayrolls.com

[6] Accessed 7/7/2023. https://ucannualwage.ucop.edu

[7] AAPM salary calculator. Accessed 3/1/2024. https://www.aapm.org/pubs/SalaryCalculator.asp

